# Continuous peripheral nerve block for in-patients with lower limb ischemic pain

**DOI:** 10.6061/clinics/2021/e2805

**Published:** 2021-05-17

**Authors:** Hermann dos Santos Fernandes, Jorge Luiz Saraiva Ximenes, Paloma Kiyomi Taguchi, Eloisa Bonetti Espada, Áquila Lopes Gouvêa, Joaquim Edson Vieira, Hazem Adel Ashmawi

**Affiliations:** Divisao de Anestesiologia, Hospital das Clinicas HCFMUSP, Faculdade de Medicina, Universidade de Sao Paulo, Sao Paulo, SP, BR

**Keywords:** Peripheral Arterial Disease, Peripheral Vascular Disease, Regional Anesthesia, Continuous Peripheral Nerve Block, Nerve Block, Analgesia, Pain Management

## Abstract

**OBJECTIVES:**

Demonstrate that continuous peripheral nerve block (CPNB) may be an alternative with adequate analgesia and a lower incidence of side effects for ischemic pain due peripheral obstructive arterial disease (POAD).

**METHODS:**

Retrospective cohort study with 21 patients with POAD, Fontaine IV graded, with foot pain. Patients were submitted to continuous sciatic nerve block (CSNB), through a perineural catheter. Primary outcomes were pain intensity (by numerical rating scale) and opioid consumption (in oral morphine equivalents).

**RESULTS:**

During CSNB, pain scores markedly decreased in comparison to the pre-block period.

**CONCLUSIONS:**

CPNB may be a good option for ischemic pain treatment in in-patients, as it provides effective pain control with fewer adverse effects.

## INTRODUCTION

Peripheral arterial obstructive disease (PAOD) is frequent among the elderly (1). Ischemic pain is the main symptom and affects the quality of life (2). Reversing the arterial obstruction is the main treatment objective, but until then, pain management is essential (3). Opioids remain in the arsenal for severe pain, but they are frequently ineffective or carry the risk of addiction and adverse effects (4).

Continuous epidural analgesia provides good analgesia, but with possible side effects and limitations of neuraxial manipulation in patients frequently receiving anticoagulant agents (5). Continuous peripheral nerve block (CPNB) may offer an advantage. As lower limb ischemic pain predominates in the foot (6), a viable option would be the approach of the sciatic nerve. There are proven benefits for continuous sciatic nerve block (CSNB) after painful orthopedic procedures (7). However, there are only few retrospective studies and case reports of this technique for the treatment of lower limb ischemic pain (8-10).

In this study, we hypothesized that CSNB may provide adequate management of lower limb ischemic pain. Its aim is to describe how pain intensity evolves with CSNB analgesia in these patients.

## MATERIALS AND METHODS

This is a retrospective cohort study of patients submitted to CSNB for ischemic pain management. The research was approved by the Hospital Ethics in Research Committee (CAAE: 55187516.4.0000.0068, N°- 1.571.917). Written informed consent was obtained from all subjects participating in this study. This article adheres to the applicable Enhancing the Quality and Transparency of Health Research checklist [The Strengthening the Reporting of Observational Studies in Epidemiology (STROBE) Statement].

Patients were recruited from August 2016 to December 2018. Twenty-one PAOD patients were enrolled. Inclusion criteria were: patients older than 18 years old, with PAOD graded as Fontaine IV (pain at rest and the presence of ulcer or gangrene), ischemic pain in the foot and who were waiting for definitive vascular surgery treatment. Exclusion criteria were: patients who did not accepted CPNB as a pain treatment option, non-cooperative patients, those with coagulation disorders, systemic or block site infection, allergies to local anesthetic and difficulty understanding the use of the patient-controlled analgesia (PCA) device, and those who refused to be submitted to peripheral nerve block.

**Figure 1:**
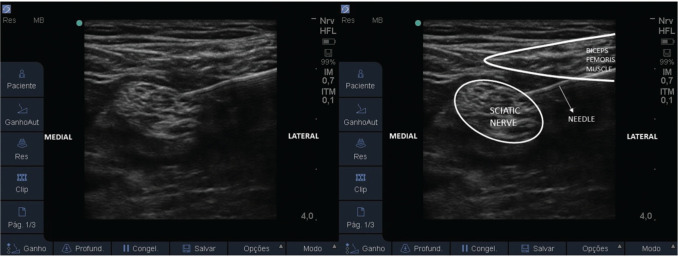
US-guided sciatic nerve block and catheter installation, popliteal approach.

Once the patient met the inclusion criteria and accepted the CPNB as a pain treatment, while the definitive surgery would not occur, baseline data were collected, and then the patient was directed for the CSNB procedure. This was performed in a procedure room, with the patient in a prone position, under noninvasive monitoring. The popliteal fossa region was cleaned with 0.5% alcoholic chlorhexidine and covered with sterile drapes. Under sterile conditions, a high frequency (8-16 MHz) linear ultrasound (US) transducer (Sonosite EDGE Portable Ultrasound System; SonoSite, Bothell, Washington) was used to locate the sciatic nerve right above its division into the tibial and common fibular nerves. After anesthesia of the skin (1% lidocaine, 3-5 mL), under US guidance, an 18G 100mm needle (Contiplex^®^ Tuohy, B. Braun, Germany) was inserted, from the lateral side of the thigh, through the biceps femoris muscle, and its tip was located close to the sciatic nerve (Figure 1). Confirmation was obtained with motor response of tibial nerve or common fibular nerve stimulation at 0.3-0.5 mA, with peripheral nerve stimulator (Stimuplex^®^ HNS 12, B. Braun, Germany). A bolus of 5 mL of ropivacaine 0.375% was injected, and an 18G epidural catheter, 50 cm long, was passed through the cannula until 3-4 cm beyond the tip of the needle. The catheter was secured to the skin with suture (Nylon 3-0 thread) and covered with transparent drapes. Another bolus of 5 mL of ropivacaine 0.375% was injected through the catheter under US visualization, for spread evaluation. A PCA pump (CADD-Legacy^®^ PCA Pump; Smiths Medical International, Ashford, Kent, UK) was connected to the perineural catheter with ropivacaine 0.2% solution, programmed as: infusion of 5-8 mL/h, bolus of 5 mL, and lock-out interval of 30 min (11). The patient was returned to the ward.

All patients were managed according to the specific institutional protocols. For pain management, all patients received a basal dose of analgesic adjuvants (dipyrone 2g, four times daily, intravenously, gabapentin 300 mg, once or twice daily, orally) and opioids, according to assisting physician’s best judgment (tramadol, codeine, methadone, oxycodone, transdermal fentanyl or morphine).

The patients kept the catheter with the PCA device until the definitive treatment of vascular surgery, but, for research purposes, data were registered for the first eight days. Primary outcomes were daily pain intensity (by numerical rating scale - NRS 0-100: 0 being no pain and 100 being the worst possible pain) and daily amount of opioid use (in oral morphine equivalents), on the day before the start of the CSNB, and for the following days under CSNB use. Patients were asked for the lowest, mean and highest pain scores, every day. Other data compared were pain reduction percentage and analgesic treatment satisfaction (by NRS 0-100: 0 total not satisfied and 100 being total satisfied). Sex, age, height, weight, associated morbidities, physical status by American Society of Anesthesiology (ASA) classification, side effects, neurological symptoms and complications were also registered.

### Statistical Analysis

Data were registered in an electronic database (REDCap 7.6.10 - © 2018 Vanderbilt University) and presented as mean±standard deviation (SD), or median (minimum - maximum) for continuous variables. Daily pain scores and opioid consumption in the CPNB period were compared to the pre-CPNB period through the Friedman test, using SPSS software version 13.0 for Windows (SPSS Inc., Chicago, IL, USA).

## RESULTS

Patients waited for vascular surgery treatment, while using CSNB, for 3-21 days. Demographic and baseline data are illustrated in Table 1.

**Figure 2:**
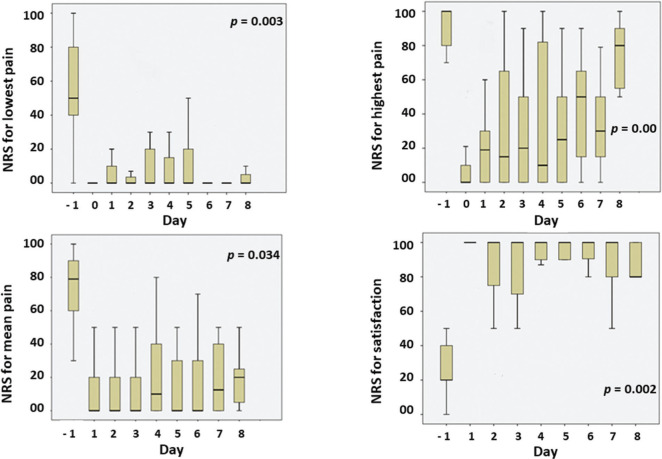
Daily evolution for lowest pain scores, highest pain scores, mean pain scores and satisfaction with analgesic treatment. NRS: numerical rating scale; Day -1: before CPNB; Day 0: immediate post CPNB initiation. Friedman test.

Pain scores and satisfaction with analgesic treatment are illustrated in Figure 2. It shows the daily evolution for the lowest, highest and mean pain scores, and daily satisfaction. Pain scores during the CPNB period were significantly lower than the pre-CPNB pain scores. Satisfaction with the treatment scores was statistically higher than pre-CPNB.

**Figure 3:**
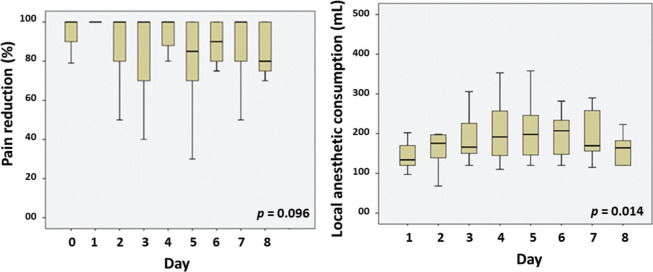
Pain reduction after the onset of CPNB and local anesthetic consumption throughout CPNB days. Day 0: immediate post CPNB initiation. Friedman test.

Pain reduction was maintained along the post-CPNB days (Friedman test *p*=0.096) (Figure 3). Local anesthetic consumption increased over time (Friedman test *p*=0.014) (Figure 3).

There was no statistically significant difference for opioid use among the different periods (Friedman test *p*=0.176).

No patients showed motor blockade, infection, catheter loss, leakage, or obstruction. Three patients reported paresthesia in the foot ipsilateral to the CSNB.

## DISCUSSION

In our hospital, PAOD patients wait several days until definitive vascular surgery treatment, due to health system demand. Providing effective analgesia becomes a challenge. Regional analgesia with an epidural catheter may be effective, but hypotension, motor blockade, and infection at the puncture site (which could lead to central nervous system infection) can occur. In this scenario, regional peripheral analgesia appears to be a viable alternative and with fewer adverse effects, as demonstrated in several postoperative studies (11). The present study corroborates these data and adds lower limb ischemic pain as an indication of CSNB, without the systemic effects that commonly occur with systemic analgesia. As most PAOD patients suffer with lower limb ischemia and foot ischemic pain, the sciatic nerve block in the popliteal fossa provides good analgesia, with minor adverse effects (12).

Opioid consumption during CSNB did not decrease on the following days. Opioids were prescribed by the assisting physicians, at fixed intervals or PRN. Therefore, even with patient’s pain improved by the CSNB, a reduction of opioid prescription did not occur because they would not risk a possible worsening of pain. As there was no increase in opioid consumption, it can be assumed that CSNB had a positive effect in analgesia.

Although analysis showed that pain scores decreased and satisfaction increased during the CSNB period, local anesthetic consumption increased. Possible explanations for these facts are minor migration of the catheter along the period, tachyphylaxis for local anesthetics, or even alternative neural pathways for ischemic pain, which would not be fully blocked by perineural blockades (13).

One important limitation of this study is in its methods. This is the retrospective cohort study. We believe that a prospective randomized controlled clinical trial would be a better type of study to compare CPNB to systemic opioids and adjuvants as analgesic treatments. Even with the theoretical concept suggesting that this may be a better option for PAOD patients (peripheral regional analgesia has a better adverse effects profile and effective locoregional action), especially in elderly, this hypothesis can only be supported by larger and better designed studies. Our group began to use this analgesic method as an attempt to provide better pain control to these patients, because systemic opioids and adjuvants had led to unsatisfactory results or intolerable side effects. Further studies are needed, including other possible benefits: better surgery results, a reduction in post-operative chronic pain and the prevention of phantom limb pain in cases of amputation.

## CONCLUSIONS

In conclusion, this study demonstrated that the use of CSNB in patients with lower limb PAOD is a viable and effective alternative for ischemic pain treatment, with a milder adverse effects profile.

**What is known**

- Peripheral obstructive arterial disease (POAD) usually evolves with severe ischemic pain in the lower limbs.
- Systemic analgesic drugs are frequently ineffective or cause adverse effects.
- Continuous peripheral nerve blocks are effective for limb pain in orthopedic surgery.

**What is new**

- Lower limb ischemic pain patients had decreased pain scores when treated with continuous peripheral nerve block.
- Continuous peripheral nerve block may be a good option for in-patient ischemic pain treatment, with a milder adverse effects profile.

## AUTHOR CONTRIBUTIONS

Fernandes HS performed the study design, patient recruitment, data collection, regional anesthesia procedures and manuscript writing. Ximenes JLS performed data collection, data analysis and manuscript writing. Taguchi PK performed data collection and manuscript writing. Espada EB performed patient recruitment and helped on study design. Gouvêa AL performed data collection. Vieira JE helped with study design, data analysis and manuscript writing. Ashmawi HA helped with study design and manuscript writing. All of the authors read and approved the final version of the manuscript.

## Figures and Tables

**Table 1:** Demographic and baseline data (before CPNB).

Number of patients (n)	21
Male (n)	12
Female (n)	9
Age (years) (mean ± SD)	61.76 ± 8.91
Body mass index (kg/cm^2^) (mean ± SD)	25.66 ± 4.01
Oral morphine equivalent daily use (mg) [median (minimum - maximum)]	90 (37-312)

## References

[1] Belch JJ, Topol EJ, Agnelli G, Bertrand M, Califf RM, Clement DL (2003). Critical issues in peripheral arterial disease detection and management: a call to action. Arch Intern Med.

[2] Aquarius AE, Denollet J, de Vries J, Hamming JF (2007). Poor health-related quality of life in patients with peripheral arterial disease: type D personality and severity of peripheral arterial disease as independent predictors. J Vasc Surg.

[3] Aronow WS (2005). Management of peripheral arterial disease. Cardiol Rev.

[4] Clair D, Shah S, Weber J (2012). Current state of diagnosis and management of critical limb ischemia. Curr Cardiol Rep.

[5] Campbell WB, Marriott S, Eve R, Mapson E, Sexton S, Thompson JF (2000). Anesthesia and analgesia for major lower limb amputation. Cardiovasc Surg.

[6] Samolsky Dekel BG, Melotti RM, Gargiulo M, Freyrie A, Stella A, Di Nino G (2010). Pain management in peripheral arterial obstructive disease: oral slow-release oxycodone versus epidural l-bupivacaine. Eur J Vasc Endovasc Surg.

[7] Ilfeld BM, Morey TE, Wang RD, Enneking FK (2002). Continuous popliteal sciatic nerve block for postoperative pain control at home: a randomized, double-blinded, placebo-controlled study. Anesthesiology.

[8] Keskinbora K, Aydinli I (2009). Perineural morphine in patients with chronic ischemic lower extremity pain: efficacy and long-term results. J Anesth.

[9] Smith BE, Fischer HB, Scott PV (1984). Continuous sciatic nerve block. Anesthesia.

[10] Hashimoto A, Ito H, Sato Y, Fujiwara Y (2013). The efficacy and safety of continuous popliteal sciatic nerve block for the relief of pain associated with critical limb ischemia: A retrospective study. Open J Anesthesiol.

[11] Ilfeld BM (2017). Continuous peripheral nerve blocks: An update of the published evidence and comparison with novel, alternative analgesic modalities. Anesth Analg.

[12] Gedikoglu M, Eker HE (2019). Ultrasound-guided popliteal sciatic nerve block: an effective alternative technique to control ischemic severe rest pain during endovascular treatment of critical limb ischemia. Pol J Radiol.

[13] Kucera TJ, Boezaart AP (2014). Regional anesthesia does not consistently block ischemic pain: two further cases and a review of the literature. Pain Med.

